# Genome analyses of the wheat yellow (stripe) rust pathogen *Puccinia striiformis* f. sp. *tritici* reveal polymorphic and haustorial expressed secreted proteins as candidate effectors

**DOI:** 10.1186/1471-2164-14-270

**Published:** 2013-04-22

**Authors:** Dario Cantu, Vanesa Segovia, Daniel MacLean, Rosemary Bayles, Xianming Chen, Sophien Kamoun, Jorge Dubcovsky, Diane GO Saunders, Cristobal Uauy

**Affiliations:** 1 Department of Viticulture & Enology, University of California Davis, Davis, USA; 2 John Innes Centre, Norwich Research Park, Norwich, UK; 3 The Sainsbury Laboratory, Norwich Research Park, Norwich, UK; 4 National Institute of Agricultural Botany, Cambridge, UK; 5 Department of Plant Pathology, Washington State University, Pullman, USA; 6 USDA-ARS, Pullman, USA; 7 Department of Plant Sciences, University of California Davis, Davis, USA; 8 Howard Hughes Medical Institute, Chevy Chase, MD, USA; 9 Gordon & Betty Moore Foundation, Palo Alto, CA, USA

## Abstract

**Background:**

Wheat yellow (stripe) rust caused by *Puccinia striiformis* f. sp. *tritici* (PST) is one of the most devastating diseases of wheat worldwide. To design effective breeding strategies that maximize the potential for durable disease resistance it is important to understand the molecular basis of PST pathogenicity. In particular, the characterisation of the structure, function and evolutionary dynamics of secreted effector proteins that are detected by host immune receptors can help guide and prioritize breeding efforts. However, to date, our knowledge of the effector repertoire of cereal rust pathogens is limited.

**Results:**

We re-sequenced genomes of four PST isolates from the US and UK to identify effector candidates and relate them to their distinct virulence profiles. First, we assessed SNP frequencies between all isolates, with heterokaryotic SNPs being over tenfold more frequent (5.29 ± 2.23 SNPs/kb) than homokaryotic SNPs (0.41 ± 0.28 SNPs/kb). Next, we implemented a bioinformatics pipeline to integrate genomics, transcriptomics, and effector-focused annotations to identify and classify effector candidates in PST. RNAseq analysis highlighted transcripts encoding secreted proteins that were significantly enriched in haustoria compared to infected tissue. The expression of 22 candidate effector genes was characterised using qRT-PCR, revealing distinct temporal expression patterns during infection in wheat. Lastly, we identified proteins that displayed non-synonymous substitutions specifically between the two UK isolates PST-87/7 and PST-08/21, which differ in virulence to two wheat varieties. By focusing on polymorphic variants enriched in haustoria, we identified five polymorphic effector candidates between PST-87/7 and PST-08/21 among 2,999 secreted proteins. These allelic variants are now a priority for functional validation as virulence/avirulence effectors in the corresponding wheat varieties.

**Conclusions:**

Integration of genomics, transcriptomics, and effector-directed annotation of PST isolates has enabled us to move beyond the single isolate-directed catalogues of effector proteins and develop a framework for mining effector proteins in closely related isolates and relate these back to their defined virulence profiles. This should ultimately lead to more comprehensive understanding of the PST pathogenesis system, an important first step towards developing more effective surveillance and management strategies for one of the most devastating pathogens of wheat.

## Background

Wheat yellow rust, also known as stripe rust, is one of the most devastating diseases of wheat worldwide. It is caused by the basidiomycete fungus *Puccinia striiformis* Westend. f. sp. *tritici* Eriks. (PST), an obligate pathogen that along with the stem (black) rust fungus *Puccinia graminis* f. sp. *tritici* (PGT) threatens worldwide wheat production [1,2]. Historically, the use of major race specific resistance (*R*) genes in wheat varieties has been an effective method for disease management. However, these approaches are hampered by the evolution of resistance-breaking races of PST. For example, the appearance of PST races that overcome widely deployed *R* genes (such as *Yr2*, *Yr9*, *Yr17* and *Yr27*) has led to destructive pandemics [3]. In recent years, concerns over yellow rust have increased with the emergence of new and more aggressive PST races that have expanded virulence profiles and are capable of adapting to warmer temperatures compared to most previous races [2]. Combined with the intrinsic ability of PST for long distance spore dispersal [4], these new races pose an increasing threat to global wheat production and food security [5].

Biotrophic plant pathogens such as rust pathogens secrete an array of proteins, known as effectors, to modulate plant innate immunity and enable parasitic infection [6]. Some of these effectors translocate inside plant cells probably through specialized infection structures known as haustoria [7-9]. Inside plant cells, effectors perturb host processes promoting pathogenesis. However, disease resistance genes in plants, known as *R* genes, encode immunoreceptors that recognize specific pathogen effector proteins. Once effector proteins are recognized, plants initiate an immune response to block the development of disease, which typically results in a localized hypersensitive reaction and programmed cell death [10,11]. The identification and characterization of these effectors and their cognate *R* genes is an important first step to understanding the wheat-PST pathosystem and consequently, to our ability to develop sustainable and potentially more durable resistance breeding strategies.

Recent availability of rust pathogen genome sequences has enabled the first steps towards wide-scale cataloguing of putative effector proteins. For instance, Saunders et al. [12] and Duplessis et al. [13] both implemented high throughput computational methods to characterize the effector complements from the fully sequenced rust fungi PGT and *Melampsora larici-populina*. Recently, Cantu et al. [14] used next-generation sequencing (NGS) to assemble a draft genome of PST isolate 130 (PST-130), annotating 22,185 putative coding sequences and classifying 1,088 of these as predicted secreted proteins. In addition, resources such as cDNA libraries generated from urediniospores and isolated haustoria (to identify PST genes specifically expressed during pathogen infection [15-18]) are publicly available. Together, they provide the necessary tools to develop a framework for characterization of the putative effector repertoire of PST.

The rapid decrease in sequencing costs now makes it possible to re-sequence multiple PST isolates to further characterize its pathogenicity arsenal. For instance, comparative genome analyses of different isolates of *Magnaporthe oryzae*, the rice blast pathogen, expanded the knowledge gained from the original reference genome considerably and helped to identify new effector genes with avirulence activity [19]. Similarly, genomic analysis of an epidemic isolate of the potato blight pathogen *Phytophthora infestans* provided insights into increased aggressiveness and virulence [20].

In this study, we re-sequenced four PST isolates with different virulence profiles and from two distinct geographical regions (the USA and the UK). We identified hetero- and homokaryotic SNPs, providing a first glimpse into PST genetic diversity on a genome wide scale. We performed independent gene discovery and annotation across all isolates to produce a combined PST secretome and identified haustoria-enriched transcripts. We validated the expression of a subset of genes during an infection time course, and revealed distinct temporal expression patterns among them. This data was then integrated using a modified version of the *in silico* pipeline described in Saunders et al. [12] to classify the putative effector repertoire of PST. Using this information, we identified putative secreted, haustoria-enriched proteins with non-synonymous polymorphisms specifically between the two UK isolates, which only differ in virulence to two known wheat differential varieties. This approach identified five effector candidates among 2,999 predicted secreted proteins that are highly expressed in haustoria and are polymorphic between the UK isolates, PST-87/7 and PST-08/21. These allelic variants are now a priority for functional validation as virulence/avirulence effectors in the corresponding wheat varieties.

## Results

### Selecting PST isolates with distinct virulence profiles

Four PST isolates from different races with distinct virulence profiles and varied geographic origin were selected for genome sequencing (Table 1). All isolates were initially identified on wheat plants, except PST-21. This isolate was originally isolated from infected triticale plants, but was subsequently shown to be virulent on wheat plants carrying the *Yr1* resistance gene [21]. The virulence profiles of the two UK isolates (PST-08/21 and PST-87/7) were examined on a set of European differential lines, complemented by a common set of Avocet ‘S’ near isogenic lines, and compared to the previously reported profiles for the US isolates (Table 1) [1,22]. The two UK isolates differed in their virulence to wheat varieties Robigus (*YrRob*) and Solstice (*YrSol*), but had common virulence to ten *Yr* genes.

**Table 1:** PST isolates used in this study and their virulence profiles

**Isolate**	**Location of isolation**	**Year**	**Natural host**	**Virulence profile on wheat**
PST-21	US	1980	Triticale	*Yr1*
PST-43	US	1990	Wheat	*Yr2*, *Yr6*, *Yr20, Yr21*
PST-130^a^	US	2007	Wheat	*Yr2*, *Yr6*, *Yr7*, *Yr8*, *Yr9*, *Yr19*, *Yr20*, *Yr21*, *Yr22*, *Yr23*
PST-87/7	UK	2003	Wheat	*Yr1, Yr2, Yr3, Yr4, Yr6, Yr7, Yr9, Yr17, Yr27, Yr32*
PST-08/21	UK	2008	Wheat	*Yr1, Yr2, Yr3, Yr4, Yr6, Yr7, Yr9, Yr17, Yr27, Yr32, YrRob, YrSol*

^a^ Previously sequenced isolate [14].

### Genome sequencing, quality assessment, and gene prediction

We used an Illumina whole-genome shotgun sequencing approach to sequence four PST isolates in addition to the recently sequenced isolate PST-130 [14]. After filtering (see Methods), the total number of PST contigs assembled ranged from 29,178 to 55,502 (Table 2). To assess the completeness of the genome sequences, we aligned the reads to the previously assembled PST-130 contigs [14]. A large proportion of the reads from the newly sequenced PST isolates aligned to the assembled PST-130 contigs (on average 86.2% SD: ±1.6%; Additional file 1), suggesting that most of the PST genome is present in the PST-130 assembly and confirming previous estimates of PST genome size [14]. In addition, a large fraction of publically available PST expressed sequenced tags (ESTs) mapped onto the assembled contigs, ranging from 81.8% in PST-87/7 to 83.2% in PST-43, further supporting a high degree of completeness of the genome assemblies. Taken together, these data suggest the US and UK isolates include between 82 and 88% of the rust genome.

**Table 2:** PST genome assembly statistics

**Isolate**	**Median coverage**	**No. of contigs**	**Total length (bp)**	**N50 (bp)**	**Max length (bp)**	**Median length (bp)**	**Average length (bp)**
PST-21	66x	43,106	73 Mb	3,960	37,006	713	1,695
PST-43	26x	49,784	71 Mb	3,264	35,154	596	1,421
PST-130^a^	59x	29,178	65 Mb	5,137	49,498	901	2,220
PST-87/7	15x	55,502	53 Mb	1,302	46,297	652	962
PST-08/21	21x	50,898	56 Mb	1,600	35,677	708	1,106

^a^ Previously sequenced isolate [14].

As an independent estimate of the degree of completeness of the assembled gene space, we implemented CEGMA analysis [23]. This protocol maps a set of 248 low copy core eukaryotic genes (CEGs) that are conserved across higher eukaryotes to the assemblies [24]. On average 72.1% (SD: ±14.6%) of the CEGs aligned as complete gene copies to the assembled contigs (Additional file 2), compared to 81.7% (SD: ± 9.7%) that aligned as fragmented partial gene copies (Additional file 2). These values are slightly lower than those of the whole genome sequence of *P. graminis* f.sp. *tritici* where 91.1% of the CEGs were mapped as complete copies and 92.7% as fragmented genes (Additional file 2). The levels of complete gene coverage were higher for all US isolates (average 83%, SD: ± 0.6%) and comparable to partial gene coverage (average 88.7%, SD: ± 0%), indicating that few core eukaryotic genes were split across contigs for these isolates. For the two UK isolates (PST-08/21 and PST-87/7) complete gene coverage was reduced (average 56.5%, SD: ± 5.7%) compared to partial gene coverage (71.3%, SD: ± 3.9%), indicating slightly higher levels of fragmentation for these genomes, which is likely attributed to the lower level of genome coverage for these isolates.

To identify open reading frames in the five assembled PST genomes, we applied the MAKER pipeline which used *ab initio* and homology based predictions and was supported by cDNA evidence generated in the course of this study (see below and Methods; [25]). On average 20,280 (SD: ±1,201) protein-coding genes were identified in the five isolates (Table 3). Over 90% of the extended CEGMA set, which includes 2,748 CEG variants from six eukaryotic genomes, matched peptides predicted in each of the assemblies, with an average of 82.4% of the protein length aligned (SD: ± 8.9%; BLASTP, e-value ≤1e^-6^). This provided an independent estimate of the completeness of the gene catalogue.

**Table 3:** Predicted gene catalogue for each PST isolate and similarity to conserved eukaryotic proteins and PGT proteins

**Species/isolate**	**No. proteins predicted**	**Median protein length (amino acids)**	**CEG matching sequences**^**a**^	**PGT matching sequences**^**a**^
PGT	20,566	266	2,596 (94.5%)	-
PST-21	20,653	211	2,626 (95.6%)	13,301 (64.7%)
PST-43	21,036	197	2,637 (96.0%)	13,313 (64.7%)
PST-130	18,149	228	2,612 (95.1%)	13,214 (64.3%)
PST-87/7	20,688	161	2,508 (91.3%)	12,801 (62.2%)
PST-08/21	20,875	172	2,592 (94.3%)	13,183 (64.1%)

^a^ BLASTP, e-value ≤ 1e^-6^. PGT, *Puccinia graminis* f. sp. *tritici* (Total PGT sequences: 20,566). CEG, Core eukaryotic genes (Total CEG sequences: 2,748).

### Estimation of diversity between PST isolates

Urediniospores constitute asexual dikaryotic spores that contain two independent nuclei. Therefore, to assess genetic variation between the two nuclei in the sequenced PST urediniospores we aligned the sequence reads of a particular isolate to the assembled contigs of the same isolate. This global analysis identified an average single nucleotide polymorphism (SNP) frequency of 5.98 ± 1.13 SNPs/kb between the two nuclei within a single isolate, referred hereafter as intra-isolate SNPs (Additional file 3).

**Figure 1:**
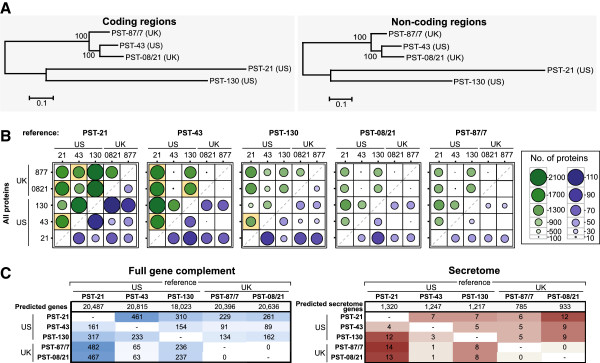
**US PST-43 may belong to the same clonal lineage as the UK isolates PST**-**87/7 and PST**-**08/21. A.** Dendrograms illustrating that US isolate PST-43 clustered with the two UK isolates PST-87/7 and PST-08/21. Dendrograms were constructed using the homokaryotic SNP information either in the coding or non-coding regions of the genome from all pair-wise alignments. **B.** Pair-wise comparison of non-synonymous mutations in synthetic gene sets illustrated that polymorphisms were more apparent when corresponding proteins representing PST-21 and PST-130 were compared against other isolates. Each gene for a given reference was taken in turn and any homokaryotic SNPs incorporated for each isolates mapped. The five genes (one reference gene and four synthetic genes) were then subjected to pair-wise polymorphism and positive selection analysis using Yn00. Circle sizes represent the number of proteins with at least one non-synonymous mutation (green circles) or under positive selection (purple circles). Pair-wise comparisons that showed enrichment in non-synonymous mutations in secreted proteins are illustrated with an orange background. **C.** The total number of genes determined as absent by mapping the sequence reads from each isolate in turn against every other isolate as a reference was greater for alignments against PST-21 and PST-130 when compared to alignments against PST-43, PST-87/7 and PST-08/21. A similar pattern was also observed when genes encoding predicted secreted proteins were assessed. Total number of genes absent from white to blue (0-482) for full gene complement and white to red (0-14) for secretome.

In addition, we aligned the reads of each isolate to the assembled contigs of the other isolates (Additional files 3 and 4). We classified these inter-isolate SNPs into two classes; heterokaryotic SNPs which refers to a variant position between the two nuclei within a single isolate that is homozygous in other isolates, and homokaryotic SNPs, which refer to homozygous variants that are polymorphic between two independent isolates (Additional file 5). On average, heterokaryotic SNPs across isolates were more frequent (5.29 ± 2.23 SNPs/kb) than homokaryotic SNPs (0.41 ± 0.28 SNPs/kb). The highest levels of diversity were found when reads of isolates PST-21 and PST-130 were mapped onto the two UK isolates (PST-87/7 and PST-08/21) and US PST-43. Here, we observed an average of 7.11 ± 1.14 SNPs/kb for heterokaryotic sites, whereas homokaryotic SNPs had a frequency of 0.64 ± 0.08 SNPs/kb. When the two UK isolates and PST-43 were compared, the heterokaryotic SNP frequency was 2.23 ± 0.53 SNPs/kb, whereas the homokaryotic SNP frequency was 0.02 ± 0.01 SNPs/kb (Additional file 3). We performed a phylogenetic analysis using the homokaryotic SNP data in both coding and non-coding regions. In the associated dendrograms, US isolate PST-43 clustered with the two UK isolates, PST-87/7 and PST-08/21 (Figure 1A). PST21 was more closely related to PST130 than to the other three isolates, and both where equally distant to the PST-43/UK cluster.

### Observed genetic diversity may reflect origin of isolates

To further characterize the genetic diversity between the five sequenced isolates of PST, we assessed the number of homokaryotic SNPs within the gene space (Additional file 6). Using this SNP data, we generated a set of four representative synthetic genes for each reference gene that incorporated the SNP information independently for each isolate to enable downstream analysis of genetic diversity (Additional file 5). Pair-wise comparisons of non-synonymous mutations in these gene sets revealed that when genes representing US isolates PST-21 and PST-130 were compared against other isolates, polymorphisms were more apparent (Figure 1B green circles; Additional file 7). For instance, when using the PST-43 genes as a reference, PST-21 showed between 1,706 and 2,047 polymorphic genes in pair-wise comparisons with all other isolates. Similarly, PST-130 showed between 1, 428 and 2,047 polymorphic genes in pair-wise comparisons with all other isolates, when using this same reference. In contrast, in pairwise comparisons between PST-43, PST-87/7 and PST-08/21 less than 130 genes were shown to be polymorphic when using the PST-43 genes as the reference (Figure 1B; Additional file 7).

Enrichment in non-synonymous mutations between genes encoding predicted secreted proteins and non-secreted proteins was assessed using the hypergeometric test. This analysis revealed enrichment in polymorphisms in secreted proteins for nine pairwise comparisons with eight originating from comparisons of PST-21 or PST-130 against other isolates (Figure 1B, orange highlighted squares). We also calculated rates of synonymous (dS) and non-synonymous (dN) substitutions for each pairwise comparison in each synthetic gene set (Additional file 8). This analysis highlighted more genes with dN/dS > 1 when PST-21 and PST-130 were compared with all other isolates, mirroring the pattern shown in the sequence polymorphism analysis (Figure 1B, purple circles). For example, for protein PST21_04206 sequence polymorphisms and positive selection were identified between the UK isolates and US PST-43 when compared to the synthetic gene from US PST-130 (Additional file 9). There was no evidence for enrichment in positive selection in genes encoding secreted proteins when compared to those encoding non-secreted proteins.

Another measure of genetic diversity is to assess the number of absent genes in pair-wise comparisons between isolates. The total number of genes classified as absent with no reads aligned, was greater for alignments against the two US isolates PST-21 (161-482 genes) and PST-130 (154-310 genes) when compared to US PST-43, UK PST-87/7 and UK PST-08/21. Less than 100 genes were shown to be absent in PST-43, PST-87/7 and PST-08/21 when alignments between these isolates were considered (Figure 1C; Additional file 10). Reciprocally, when PST-21 or PST-130 sequence reads were mapped against the genome assemblies of the other three isolates (PST-43, PST-87/7 and PST-08/21), a greater number of genes were noted as absent when compared to the alignment of the other three isolates (Figure 1C; Additional file 10). A similar pattern was also observed when genes encoding predicted secreted proteins were assessed (Figure 1C).

This analysis confirmed our previous observation that US PST-130 and PST-21 appear more genetically diverse when compared to other isolates in this study, potentially reflecting different evolutionary origins for these isolates. The observed genetic diversity for US PST-21 may reflect adaptation to a different host, triticale, when compared to isolates in this study that were isolated specifically from wheat.

### RNA-seq analysis identified transcripts specifically enriched in haustoria

**Figure 2:**
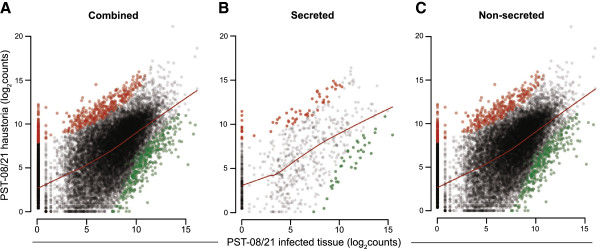
**Comparison between infected tissue and isolated haustoria RNAseq libraries.** Scatter plot of log_2_ transformed sequencing counts generated by aligning RNAseq reads to all PST-08/21 genes (**A**), those that encode predicted secreted peptides (**B**), or encode non-secreted peptides (**C**). Red and green colored circles correspond respectively to transcripts that were identified as significantly enriched or depleted in isolated haustoria as determined by DESeq analysis (*P* ≤ 0.01). Red lines represent the locally weighted polynomial regression (LOWESS method).

We performed RNAseq analysis of UK PST-08/21 infected wheat leaves and isolated haustoria to identify haustoria-enriched transcripts (Additional file 11). After filtering, we aligned reads to the PST-08/21 assembly and also generated *de novo* assemblies that were used to support the gene prediction pipeline. We conducted differential expression analysis after normalization, using DESeq to identify genes that were significantly up-regulated (False discovery rate <0.01) in haustoria compared to infected tissue (Figure 2A; Additional file 12). Within the subset of transcripts encoding for predicted secreted proteins (933 genes, see Methods), we identified 57 genes that were significantly enriched in haustoria (6.1%), compared to 31 (3.3%) that were significantly depleted (Figure 2B). A much lower proportion of genes encoding for non-secreted proteins was identified as enriched (2.1%; 411 of 19,703 genes) or depleted (1.7%) using the same analysis (Figure 2C). These results suggest that this approach is effective in selectively enriching for secreted proteins, which are likely to be haustorial-expressed transcripts.

### Defining and classifying the effector repertoire of PST

**Figure 3:**
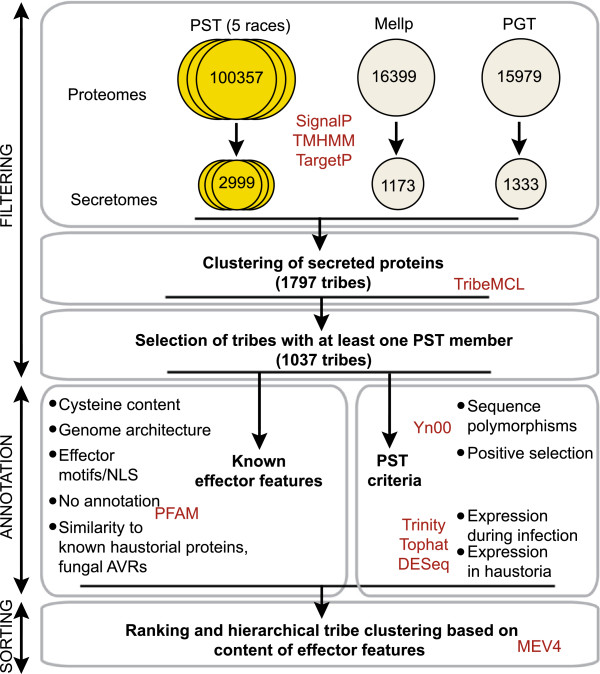
**Clustering of secreted proteins and annotation of protein tribes based on known effector features and PST**-**specific criteria.** A bioinformatic pipeline was implemented to identify groups of secreted proteins with characteristic effector features. The proteomes of the five PST isolates were combined (totaling 100357 proteins), 5502 secreted proteins predicted, and redundancy reduced. The consolidated PST secretome (2999 proteins) was combined with predicted secretomes from *P. graminis* f. sp. *tritici* (PGT) and *Melampsora larcia populina* (Mellp) and proteins grouped based on sequence similarity (Markov clustering). Tribes containing at least one PST member (1037 tribes) were annotated with known effector features or PST-specific criteria. Finally, tribes were ranked and heirarchical clustering implemented based on their content of proteins with known effector features. Programs are indicated in red. NLS, nuclear localization signal.

To identify and classify candidate effectors from PST, we implemented a modified version of the bioinformatics pipeline described in Saunders et al. [12] (Figure 3). The five PST proteomes were combined (totaling 100,357 proteins), 5,502 secreted proteins predicted, and redundancy in the combined secretome reduced (see Methods). After consolidation, a total of 2,999 predicted secreted proteins were selected representing the diversity of the five PST secretomes (Figure 3). To enable the identification of any potential conserved rust effectors, secreted proteins were also classified and incorporated in this study from the proteomes of *Puccinia graminis* f. sp. *tritici* and *Melampsora larici-populina*, comprising 1,333 and 1,173 secreted proteins, respectively. The three rust pathogen secretomes were combined and grouped into 1,797 protein tribes based on sequence similarity using Markov clustering [26]. The final filtering step of the bioinformatics pipeline reduced the total to 1,037 tribes, each containing at least one PST secreted protein. Proteins in the 1,037 PST-containing tribes were then annotated with both known effector features from other pathosystems and PST-specific criteria (Figure 3). The later criteria focused on allelic variation between the five PST isolates and the expression of genes at the plant-pathogen interface as determined by the RNA-seq analysis of PST-08/21-infected wheat leaves and isolated haustoria. Expression of genes either during infection or specifically in haustoria was then added as criterion in the effector-mining pipeline (Figure 3).

### Identifying candidate effectors of high interest

**Figure 4:**
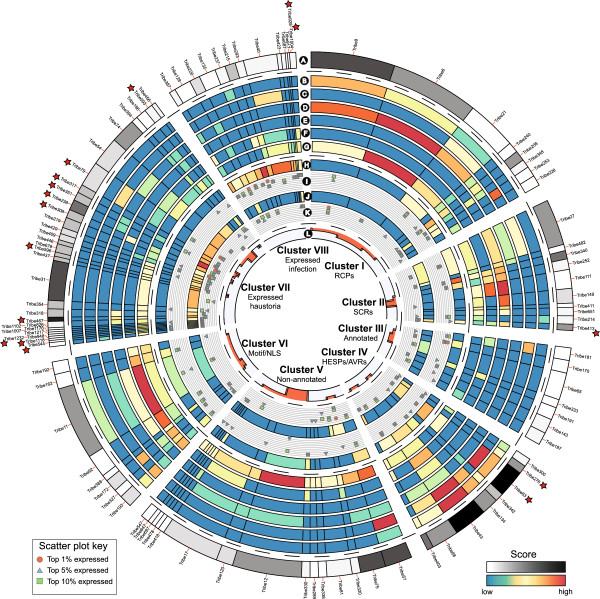
**The top 100 ranked protein tribes containing putative effector candidates.** Clusters were determined using hierarchical clustering of the top 100 ranked tribes containing putative effector candidates. **A.** Combined score used to rank tribes based on their content of effector features. **B.** Score for similarity of tribe members to haustorial expressed secreted proteins (HESPs) or characterized fungal AVRs. **C.** Score for number of members encoded by genes with at least one flanking intergenic region >10 Kb. **D.** Score for number of members classified as repeat containing (RCPs). **E.** Score reflecting number of members classified as small and cysteine rich (SCRs). **F.** Score for number of members containing any characterized effector motifs or nuclear localization signals (NLS). **G.** Score for number of members not annotated by PFAM domain searches. **H.** Score for number of members in the top 100 expressed in infected material, determined by mRNA-seq analysis. **I.** Scatter plot indicating number of members in the top 1, 5 or 10% expressed in infected material. **J.** Score for number of members in the top 100 expressed in haustoria, determined by mRNA-seq analysis. **K.** Scatter plot indicating number of members in the top 1, 5 or 10% expressed in haustoria. **L.** Number of PST members showing sequence polymorphisms between isolates. Stars indicate tribes that contain members assessed for expression using qRT-PCR.

To order and cluster protein tribes based on known effector features and PST-specific annotation we implemented the sorting module of the pipeline (see Methods; Figure 3). This resulted in overall scores for each tribe that reflected their likelihood of containing potential effector proteins (Additional file 13). The features associated with the top 100 ranked tribes are displayed in Figure 4.

**Figure 5:**
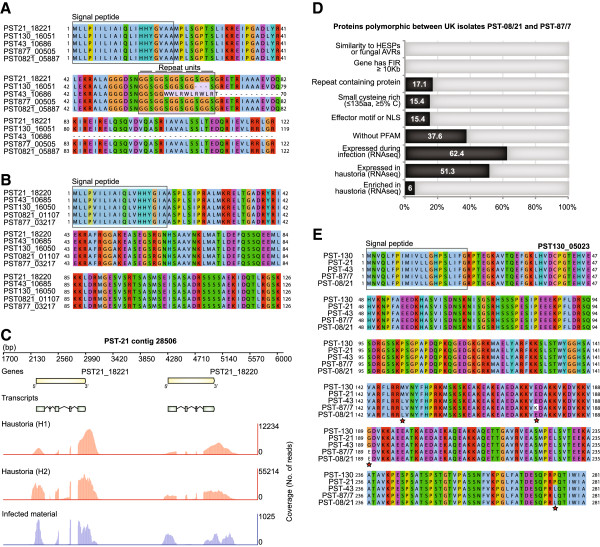
**Secreted proteins with high likelihood as candidate effectors. A.** Sequence alignment of PST21_18220, a member of tribe 238, and the corresponding alleles from the other four isolates illustrating sequence polymorphisms specifically between the US isolates, PST-21, PST-43 and PST-130. **B.** Sequence alignment of the second member of tribe 238, PST21_18221, and its alleles from other isolates illustrating that this protein was highly conserved across isolates. **C.** The two members of tribe 238, PST21_18220 and PST21_18221, are in close proximity within a single contig in the genome sequence. The corresponding genes were expressed during infection and were also highly expressed and enriched in haustorial samples as determined by mRNA-seq analysis. **D.** Features displayed by the 117 proteins that were identified as polymorphic between the two UK isolates PST-08/21 and PST-87/7. **E.** Sequence alignment of PST130_05023 and the synthetic genes that incorporate the SNP information from the other four isolates sequenced, illustrating sequence polymorphisms between isolates. Polymorphic residues are indicated below the sequence by red stars.

To select proteins with a high likelihood as candidate effectors we focused on tribes that ranked highly based on our scoring system, are highly expressed and enriched in haustoria, and display sequence polymorphisms between isolates. For example, Tribe 238 is ranked 10^th^ in our scoring system and is a member of Cluster VII, which reflects the fact that both members, PST21_18221 and PST21_18220, are expressed highly in haustoria and are significantly enriched in haustoria with respect to infected tissue (*P* = 0.001, 31 and 42-fold, respectively). This tribe represents two distinct proteins that are present in all isolates sequenced in this study. One protein was identified as polymorphic specifically between the US isolates, whereas the second protein was conserved across all isolates (Figure 5A-B). The two proteins are encoded by genes within close proximity on a single contig in the sequenced genomes (Figure 5C). This suggests that these genes could have arisen from a duplication event, which is further supported by sequence similarity between the two proteins within the N-terminus. This is consistent with the concept that pathogenicity factors may arise from gene duplication events followed by rapid diversification to evolve new functions [27].

### Candidate effectors with sequence polymorphisms between the UK isolates PST-08/21 and PST-87/7

We identified secreted proteins that displayed polymorphisms specifically between the two UK isolates PST-87/7 and PST-08/21, which only differ in virulence to two wheat differential varieties Robigus (*YrRob*) and Solstice (*YrSol*). The properties of the 117 proteins identified are displayed in Figure 5D. Of these 117 proteins, 14 were members of tribes that ranked in the top 100 in our scoring system and five were encoded by genes which were significantly enriched and in the top 10% of expressed transcripts in both haustorial samples (Table 4). For all five genes a single amino acid substitution was evident between the sequences for the two UK isolates. For example, for PST130_05023, four amino acid substitutions were identified; one differential between the UK and US isolates, two between UK isolates and US PST-43 when compared to the other US isolates, and one specific substitution between the two UK isolates (Figure 5E). Focusing on proteins that display polymorphisms between these two isolates may facilitate the detection of avirulence and corresponding virulence effector proteins that are specifically differentially recognized by *YrRob* and *YrSol*.

**Table 4:** Secreted proteins with non-synonymous substitutions between UK isolates PST-87/7 and PST-08/21 and in tribes ranking within the top 100 potential effector tribes

**Gene ID**	**Tribe no.**	**Tribe ranking**	**Length (amino acids)**	**Similarity to HESPs or fungal AVRs**	**1 FIR ≥10 Kb**	**No. of repeat units**	**SCR protein**	**Effector motifs (amino acid position)**	**NLS signal**	**PFAM mapping**	**Infected material (reads)**	**Haust. 1 (reads)**	**Haust. 2 (reads)**	**Enrichment in haustoria**	
														**Fold change (log2)**	***P *value (adj)**
PST130_14637	9	6	609	No	No	10	No	-	No	No	0	0	4	1.02	0.68
PST21_19014	11	14	167	No	No	0	No	Y/F/WxC(85); LIAR(32)	No	Yes	535	120	534	-0.38	0.87
PST887_17743	11	14	185	No	No	0	No	Y/F/WxC(103); LIAR(32)	No	Yes	904	167	695	-0.61	0.70
PST130_00418	8	17	176	No	No	20	No	-	No	No	6	0	361	4.15	1.00
PST21_12116	74	20	455	No	No	0	No	-	No	Yes	298	3708^b^	34272^b^	5.99	<0.001
PST130_05023	351	22	281	No	No	6	No	-	No	Yes	2475^a^	7368^b^	62158^c^	3.85	0.011
PST21_18360	437	23	394	No	No	0	No	-	No	No	480	5957^b^	15413^b^	5.08	0.001
PST130_00285	317	25	207	No	No	0	No	-	No	Yes	919	6699^b^	36240^b^	4.77	0.001
PST21_15642	308	28	102	No	No	0	Yes	-	No	No	209	6002^b^	6286^a^	5.96	<0.001
PST21_17946	17	39	177	No	No	3	No	-	No	No	396	417	3015	2.21	0.19
PST21_04206	456	58	515	No	No	0	No	-	No	Yes	2563^a^	513	1806	-0.70	0.65
PST21_20471	21	83	376	No	No	13	No	-	No	No	24	7	34	0.05	0.69
PST43_15488	111	94	94	No	No	0	Yes	Y/F/WxC(23)	No	Yes	399	0	76	-3.81	0.17
PST21_15274	111	94	91	No	No	0	Yes	Y/F/WxC(23)	No	No	64	1	24	-2.46	0.17

HESPs, Haustorial expressed secreted proteins. FIR, flanking intergenic region. SCR, small cysteine-rich. NLS, nuclear-localization signal. Haust, Haustorial library.

Genes expressed in the top 10 (a), 5 (b) and 1% (c) in a particular RNAseq dataset.

### Transcript profiling reveals peaks in gene expression for candidate effectors during infection

**Figure 6:**
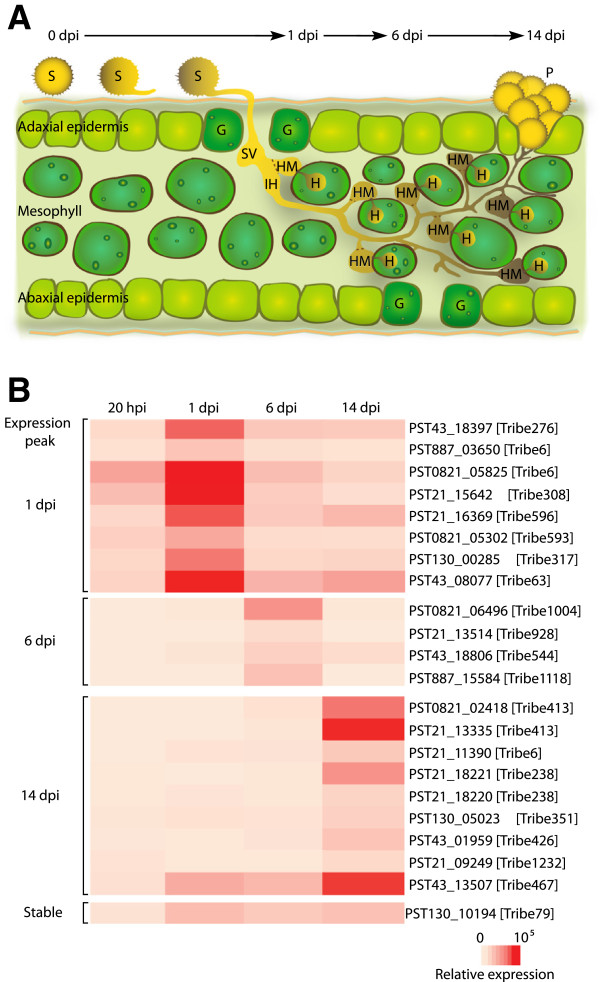
**Quantitative RT**-**PCR revealed peaks of expression for the selected effector candidates during plant infection. A.** Schematic representation of the stages of PST development during plant infection. **B.** Quantitative RT-PCR was undertaken at four stages of PST-08/21 infection for a subset of 22 effector candidates. Three peaks of expression were noted at 1 day post-inoculation (dpi), 6 dpi and 14 dpi. hpi, hours post-inoculation; S, uredinospore; SV, substomatal vesicle; IH, invasive hyphae; HM, haustorial mother cell; H, haustorium; P, pustule; G, guard cell.

To further characterize a subset of candidate effectors, we assessed their expression profiles using quantitative RT-PCR across different infection time points (20 hours and 1, 6 and 14 days post inoculation (dpi)) (Figure 6A; Additional file 14). Twenty-two putative effectors representing nineteen tribes were selected based on their rank as likely effectors and their expression levels determined by mRNAseq analysis of isolated haustoria (Table 5). The gene models of these effector candidates were further validated during the qRT-PCR analysis using primers that spanned the splice site junctions.

**Table 5:** Tribe members selected for expression profiling during PST infection of wheat

**Tribe no.**	**Tribe ranking**	**No. of proteins**	**Average protein length (amino acids)**	**Species distribution (No. of proteins)**	**No. of proteins in top 100 expressed during infection**	**No. of proteins in top 100 expressed in haustoria**	**Members assessed for peak in expression**	**General effector features (No. of proteins)**
63	2	13	180	PST (2), PGT (5), Mel (6)	2	2	1 dpi (1)	Similarity to *M. lini* hesp-C49 (10); Internal repeats (2); Members encoded by genes with 1 FIR ≥10Kb (2); Without PFAM annotation (11)
467	3	3	109	PST	0	3	14 dpi (1)	SCRs (3); Without PFAM annotation (3)
238	10	5	112	PST	0	5	14 dpi (2)	Internal repeats (2)
413	12	3	95	PST (2), PGT (1)	1	2	14 dpi (2)	Y/F/WxC (2); SCRs (3); Without PFAM annotation (1)
276	13	4	288.5	PST (2), PGT (1), Mel (1)	0	2	1 dpi (1)	Similarity to *M. lini* hesp-735 (3) and hesp-379 (1); Internal repeats (1); Without PFAM annotation (1)
351	22	4	276.5	PST (3), PGT (1)	0	3	14 dpi (1)	Internal repeats (2); Without PFAM annotation (2)
317	25	4	209.5	PST (3), PGT (1)	0	3	1 dpi (1)	-
308	28	4	145	PST	0	3	1 dpi (1)	SCRs (3); Without PFAM annotation (3)
596	31	2	139	PST	0	2	1 dpi (1)	Without PFAM annotation (2)
79	34	12	299.5	PST (8), PGT (1), Mel (3)	0	4	Stable (1)	Y/F/WxC (2), LIAR (1); Members encoded by genes with 1 FIR ≥10Kb (1); Without PFAM annotation (2)
928	43	1	163	PST	1	1	6 dpi (1)	Y/F/WxC (1); Without PFAM annotation (1)
1004	43	1	132	PST	1	1	6 dpi (1)	Without PFAM annotation (1)
593	60	2	160	PST	0	1	1 dpi (1)	Without PFAM annotation (1)
544	75	2	216	PST (1), PGT (1)	0	1	6 dpi (1)	SCRs (1); Internal repeats (1); Without PFAM annotation (2)
1232	78	1	163	PST	0	1	14 dpi (1)	Without PFAM annotation (1)
1118	96	1	201	PST	0	1	6 dpi (1)	LIAR (1)
426	133	3	142	PST	1	1	14 dpi (1)	Without PFAM annotation (1)
6	154	49	224	PST (27), PGT (21), Mel (1)	0	4	1 dpi (2), 14 dpi (1)	Y/F/WxC (1), LIAR (2), RXLR (1); Without PFAM annotation (31)

dpi, days post-inoculation.

Three peaks in expression were observed for the candidate effectors using qRT-PCR, with eight more highly expressed at 1 dpi, four at 6 dpi and nine at 14 dpi (Figure 6B). Haustoria are already formed at 1 dpi [28] suggesting that some of these candidate effectors might be involved in the very early stages of infection. Two of the five haustoria-enriched polymorphic genes between the UK isolates were amongst those most highly expressed at 1 dpi (PST21_15642 and PST130_00285), whereas one showed highest expression at 14 dpi (PST130_05023). Only one candidate effector (PST130_10194) was stably expressed at all time-points assessed during infection. For tribes 238, 413 and 6, the expression profiles of several members of each tribe were assessed. With the exception of one member of tribe 6 (PST21_11390), members of the same tribe tended to peak in expression at the same time-point, indicating that they may be involved at similar stages during disease progression. These results are consistent with the concept of sequential waves of expression of different sets of effectors during the infection process [29].

## Discussion

### Polymorphic secreted proteins as effector candidates

In this study, we re-sequenced four PST isolates to identify effector candidates and relate them to their distinct virulence profiles. Once the complete effector complement was defined, we focused on highlighting polymorphic effector candidates that may reflect rapid adaptation to specific host targets, such as disease-resistance proteins amongst others. The list of effector candidates we developed here should prove useful to the rust research community to initiate functional screens for effector activity. One possible strategy would be the induction of hypersensitive cell death by potential avirulence proteins (AVR) on differential wheat lines using an effector delivery system as is routinely conducted in the potato-*P. infestans* pathosystem [30].

The co-evolutionary arms race between pathogens and plants has led pathogens to respond by mutating or losing AVR effector proteins [31]. A survey of six *Melampsora lini* isolates identified diversifying selection acting on the *Avr567* locus, with substitutions identified in surface exposed residues that dictated host recognition [32,33]. Likewise, Yoshida et al. [19] identified three novel *Avr* genes that were absent from the *M. oryzae* genome sequence (isolate 70-15) but present in the re-sequenced avirulent isolate Ina168. In PST, we did not find evidence of large scale absence of genes or putative effectors on the scale identified in *M. oryzae*[19]. Even when comparing between the most distantly related isolates, we found less than 15 predicted secreted protein genes to be absent in any pairwise comparisons (<1.3% of the total predicted secretome, Figure 1C). Although this is a first and limited sampling of the available PST diversity, it suggests that gene loss may not be the main driver for changes in virulence. Therefore, assessing allelic variation in putative effectors between distinct PST isolates will likely be important to identify avirulence/virulence alleles.

### Uncovering the evolutionary origin of PST isolates

Comparative genomic studies are powerful tools for assessing the evolutionary origin of particular races. In this study, the limited genetic diversity found between US isolate PST-43 and the two UK isolates, PST-87/7 and PST-08/21, could be indicative of these isolates belonging to the same clonal lineage. This is supported by a diversity study based on 117 amplified fragment length polymorphism (AFLP) fragments which showed that PST isolates collected before 2000 in the US and Europe clustered together [34], thereby suggesting a common origin. Interestingly, the two UK isolates used in this study were collected after 2000 (2003 and 2008), yet still cluster with the older US race. This agrees with virulence data which suggests that despite the emergence of new highly aggressive isolates after 2000 in some European countries (Denmark and Sweden), these have not yet appeared in UK fields (unpublished observations, Rosemary Bayles). Conversely, differences between races can reflect diverse evolutionary origins or host specificity. In this study, US isolates PST-130 and PST-21 appear more genetically diverse when compared to the other isolates we sequenced. This is supported by studies showing that US races isolated post-2000, such as PST-130, may have a different origin than those isolated pre-2000 [5,34] and that PST-21 displays host specificity for triticale [21]. The data generated is not only valuable for addressing these evolutionary questions, but can also be used by the wider rust research community for development of more extensive polymorphic markers for large-scale screening of PST field populations and to complement the current set of dominant AFLP markers.

### Utility of haustoria-enriched transcripts in effector mining

As biotrophic fungi and oomycetes secrete effectors from highly specialized structures, known as haustoria, we also undertook transcriptome analyses of infected tissues and isolated PST haustoria to identify potential *Avr* genes. For example, sequencing ESTs from *M. lini* isolated haustoria led to the identification of three uncharacterized *Avr* genes [7], suggesting that haustorial expressed transcripts represent a rich source for *Avr* effector identification. To identify haustoria enriched transcripts, we compared gene expression levels between infected material and isolated haustoria. Although RNAseq analysis of infected material was limited to a single biological replicate, we used the DESeq approach [35] to assess enrichment as this package is suited for working with partial replicates [36]. This enabled us to estimate levels of variation in the infected material sample based on that observed in the replicated haustoria samples. Despite the low power of detection with this approach we could identify haustoria enriched genes with sufficient confidence. These transcriptome results helped to further classify and prioritize the PST secretome. It also establishes a robust baseline from which to extend this analysis to other isolates.

### Improving existing PST gene models

An additional contribution of the expression studies was to generate more reliable PST gene models, which is critical to identify genes encoding for proteins with secretion signal peptide sequences. This was especially relevant for the downstream effector prediction pipeline since this constitutes the first criteria with which proteins are classified and filtered. We addressed this potential limitation by firstly complementing the homology based and *ab initio* prediction pipeline [25] with the *de novo* transcript assemblies generated from the RNAseq data and secondly, by integrating independent gene predictions from all isolates. Whilst assessing the independent gene sets obtained for the different isolates we noted that for many fragmented genes, a non-fragmented copy could be identified in at least one genome. Therefore, it is anticipated that the gene catalog presented here will be a better representation of the PST gene complement, than the previous one which was based on genomic sequences alone [14]. The overall accuracy of the gene predictions herein were also supported by the identification of higher frequencies in nucleotide variants in the intronic regions than in the exons for both heterokaryotic and homokaryotic SNPs (Additional file 3).

### Applying comparative genomics to effector mining

The recent release of draft genome sequences for several rust pathogens has provided the first step towards wide-scale cataloguing of putative effector proteins [13,14]. So far, these studies have been limited to secretome prediction and annotation from a single reference genome. Here, we employed a comparative genomics approach to move beyond the single isolate-directed catalogue and utilise the distinct virulence profiles of the isolates sequenced to identify putative virulence/avirulence effectors. Our aim was to integrate genomics, transcriptomics and effector-focused annotation to generate a rich source of information that could be utilized to identify effector candidates in PST. However, this raises the challenge of developing methods that can utilize the vast abundance of data to address clearly defined biological questions. The clustering and classification methods used here allowed us to organize the complexity of these large datasets. Providing the data in an easily comprehensible format (Figure 4) will enhance accessibility and help the wider rust research community to rapidly access effector candidates. Moreover, this method provides a logical framework to prioritize candidate effector genes for functional validation, an approach that will become increasingly powerful as additional races are re-sequenced, more mRNAseq data becomes available and the avirulence activity of candidate effectors is established.

The use of association analysis to identify candidate avirulence proteins has been successfully implemented in other pathosystems [19,37]. As a first step in this direction, we examined two UK isolates (PST-87/7 and 08/21) that share common virulence for ten wheat *Yr* genes (*Yr1*, *Yr2*, *Yr3*, *Yr4*, *Yr6*, *Yr7*, *Yr9*, *Yr17*, *Yr27*, *Yr32*), but differ in their ability to infect two UK varieties, Robigus (*YrRob*) and Solstice (*YrSol*). We found no evidence of gene loss between these isolates. However, focusing on polymorphic variants that were also highly expressed and enriched in haustoria (within the top 10% expressed genes) reduced the dataset from 2,999 secreted proteins to just five polymorphic effector candidates. These genes and their allelic variants are now a priority for functional validation as virulence/avirulence effectors in the wheat varieties Robigus and Solstice.

### The next challenge – functional validation of effector candidates

The next step will be to establish functional validation methods for rust effectors in wheat. This crucial phase is still in its infancy [38,39] and will most likely be limited to testing a handful of candidate genes in the initial stages. Several groups have attempted to modify existing heterologous expression systems from other pathosystems to establish a reliable method for testing rust AVR effector candidates in wheat. This includes delivery of effector candidates directly into wheat cells by expressing them in bacterial pathogens for delivery by the type III secretion system [39] or through virus-mediated approaches [40]. Alternatively, host-induced gene silencing could be utilized to transiently silence and test PST pathogenicity or virulence genes. Recently, silencing of three endogenous genes in *Puccinia triticina*, the wheat leaf rust pathogen, was reported using transient *Agrobacterium*-mediated expression of corresponding RNAi constructs in wheat [38]. If established as a large-scale functional assay system, transient expression could be utilized to enable high-throughput loss-of-function screening of a diverse array of PST effector candidates in wheat.

## Conclusions

This study provides valuable information including (i) an estimate of the distribution of genetic diversity within and among PST isolates, (ii) characterization of the expression of PST genes in infected tissue and haustoria using RNAseq analysis, (iii) a bioinformatics pipeline to organize and prioritize candidate effector genes for functional studies and (iv) a list of candidate avirulence genes which display polymorphisms specifically between two UK isolates. As additional races and divergent isolates are re-sequenced, polymorphic effectors will be more easily distinguished from underlying genetic diversity between isolates, streamlining the cataloging of potential avirulence/virulence proteins for testing. The functional validation of these will ultimately lead to a more comprehensive understanding of the PST pathogenesis system, an important step towards developing more effective surveillance and management strategies for one of the most devastating pathogens of wheat.

## Methods

### Genome sequencing, assembly and gene prediction

Genomic DNA was extracted for each isolate from dried urediniospores using the CTAB method as described by Chen et al. [41]. DNA libraries were prepared as described previously for PST-130 [14]. Library quality was confirmed before sequencing using the Agilent 2100 Bioanalyzer (Agilent Technologies, UK). Sequencing was carried out on an Illumina HiSeq machine at the DNA Technologies Service core at UC Davis. Adapter and barcode trimming and quality filtering were carried out using the FASTX-Toolkit [42]. Terminal nucleotides at the 3′ end with sequencing quality below Q20 were removed and reads that after trimming were shorter than 40 nucleotides were discarded. FASTQ files of high-quality trimmed sequences were used for downstream analysis. The pair-end trimmed and filtered reads were *de novo* assembled using the CLC Genomic Workbench 4.0 software [43]. The following parameters were applied: mismatch, insert, and deletion cost = 3; length fraction = 0.3; similarity = 1.0 no global alignment; conflict resolution = vote; ignore nonspecific matches; min contig length = 300 bp; paired-end distance = 100–600 bp. Contigs with homology with non-fungal sequences in the complete NCBI nt collection were considered contaminant and discarded. Assemblies were deposited at GenBank and the SRA and WGS accession numbers are listed in Additional file 1 together with the general sequencing and assembly metrics. To assess genome completeness reads from each isolate were filtered for contaminants and then mapped to the assembled PST-130 contigs. PST ESTs used for assessment of genome completeness were obtained from GenBank. Gene prediction was undertaken following the MAKER pipeline [25] using PST ESTs, *de novo* transcript assemblies generated in this work (see below), and PGT and *P. triticina* (PT) peptide sequences as templates for gene model discovery. The homology based gene prediction implemented in MAKER was integrated with the *ab initio* prediction program SNAP [44] using as training dataset the above mentioned PST, PGT, and PT datasets.

### Assessing genetic diversity

Illumina pair-end genomic sequence reads from each isolate were mapped onto each other isolate used as a reference using Novoalign (version 2.07.18 [45]; parameters used: -R99, -i PE 500,400 -Q30 -o SAM). Custom perl scripts were used to extract the mapping counts from the SAM files and determine the sequencing coverage of each gene. Mapping metrics are reported in Additional file 4. Genes were classified as absent when no reads mapped to the coding region of a particular gene. SAM files were converted into BAM format, sorted, indexed, and analyzed using Picard tools (version 1.55 [46]). Single nucleotide polymorphisms (SNPs) were determined using the Genome Analyzer Toolkit pipeline (GATK, version 1.65; [47]). The GATK RealignerTargetCreator and IndelRealigner programs were applied to realign the reads mapped on indel sites. The GATK UnifiedGenotyper with parameters --outputmode EMIT_VARIANT_ONLY and -glm SNP was then used to identify SNPs. The alignment of sequencing reads to the correct genomic location is critical for the accurate identification of genetic variants. Thus, the variant calling was restricted to those sites that did not display either too low (minimum 5x coverage) or too high coverage. Genomic regions that display a higher than expected number of aligned reads are likely to be stretches of similar or repetitive sequences that have been assembled together [14]. To reduce the number of false positive identified (i.e. SNPs between non-orthologous region), we imposed a maximum coverage threshold equivalent to 1.5 times the median coverage over the entire assembly. To determine whether the calculated median coverage is a valid proxy of the coverage associated with single copy genes, sequencing reads were remapped on a set of 10 single copy genes previously identified in the PST-130 assemblies [14]. The sequencing coverage on these single copy genes was very similar to the median coverage over the entire assembly [e.g. 63.9x coverage (SD: ±6.7) and 59.9x (SD: ±5.7) in PST-21 and PST-130, respectively]. Assuming that the coverage of a repetitive region increases proportionally with the copy number, the maximum coverage threshold we selected is expected to reduce both Type I and Type II errors of calling a single copy locus to less than 0.001 for all races. Heterokaryotic sites were identified as sites with allelic frequency = 0.5. If a site in the reference had allelic frequency of 0.5 or the mapped reads from another isolate had allelic frequency = 0.5 the site was considered heterokaryotic (Additional File 5). Homokaryotic variants were identified as sites that are homokaryon both in the reference (allelic frequency = 1.0) and in the mapped reads (frequency of the alternative allele = 1.0). The overall ratio of transition over transversion mutations across all five isolates was 2.30 ± 0.17. These values are consistent with human studies [48] and as expected, are higher than the 0.5 ratio that would be obtained if all substitutions were equally probable.

To assess genetic diversity between isolates synthetic gene sets were generated. Each gene for a given reference was taken in turn and any homozygous SNPs incorporated for each isolate mapped (Additional File 5). The five genes (one reference gene and four synthetic copies) were then subjected to pair-wise polymorphism and positive selection analysis using the bioinformatics program Yn00 [49]. Any pair-wise comparisons that yielded a dN value > 0 were classified as polymorphic and those with dN/dS values > 1 were classified as under positive selection. The polymorphism and positive selection analysis was automated using custom Perl scripts.

### Preparation of PST infected plant material for mRNAseq analysis

Two sample types were selected for mRNAseq analysis, infected wheat leaf material and purified haustoria. Wheat seedlings (cv Avocet ‘S’) were infected with PST-08/21 and incubated in the dark at 10&z.ousco;C with high relative humidity for 24 hours. Plants were then transferred to a 16 h/8 h day/night cycle at 18&z.ousco;C. For infected leaf samples material was collected at 6 and 14 days post-inoculation (dpi) and pooled prior to RNA extraction. Alternatively, haustoria were isolated from infected leaf material 7 dpi. Two independent haustorial isolations were undertaken, each from ~2,500 infected wheat seedlings. A total of 20 g of infected leaf segments were collected that displayed localized infection, as determined by small white flecks without rust sporulation. The segments were washed with 70% ethanol for 1 min, followed by water for 1 min, then haustoria isolation performed as described in Song et al. [50] using either three (H1) or two (H2) rounds of sucrose gradient purification.

Total RNA was extracted from both the infected wheat leaf material and purified haustoria using TRIzol reagent (Invitrogen, UK) and treated with DNA™-free DNase (Manufacturer) and the Removal Reagents kit (Ambion, UK) following the manufacturer’s instructions. The quantity and quality of RNA extracted was assessed using the Agilent 2100 Bioanalyzer (Agilent Technologies, UK).

### Transcriptome sequencing, *de novo* assembly and alignment

cDNA libraries were prepared using the Illumina TruSeq RNA Sample preparation Kit (Illumina, US) for RNA extracted from infected material and the haustoria H2 sample, whereas RNA extracted from the haustoria H1 sample was processed using the Clontech SMARTer Ultra Low Input RNA Kit (Clontech, Takara Bio Europe, France). Library quality was confirmed before sequencing using the Agilent 2100 Bioanalyzer (Agilent Technologies, UK). Sequencing was carried out on an Illumina Genome Analyzer II at The Sainsbury Laboratory. The 76 bp pair-end reads were filtered for quality as described above and aligned to genomic PST assemblies using Bowtie (version 0.12.7 [51]) in global alignment mode allowing a maximum of two mismatches (parameters: -v 2 --best -k 1). SAM output files were parsed with custom Perl scripts to determine the number of reads mapping to a single PST transcript. DEseq [35] was used to normalize raw transcript counts and to compare libraries from PST infected tissue and enriched haustoria preparations in order to determine potential transcript enrichment in isolated haustoria compared to whole infected material (version 1.10.1). The reads were also assembled *de novo* using the Trinity package [52] with default settings to serve as additional evidence for gene prediction. The Illumina reads were deposited in the National Center for Biotechnology Information’s Gene Expression Omnibus (GEO) and are accessible through GEO (GSE42496; [53]).

### Secretome prediction and Markov clustering

The predicted proteomes of all five PST isolates were combined and secreted proteins predicted using SignalP2 with parameters described in [54]. Transmembrane domain containing proteins and proteins with mitochondrial signal peptides were removed using TMHMM [55] and TargetP [56], respectively. To reduce redundancy secreted proteins were clustered that displayed 99% sequence identity over 50% of the sequence length, using CD-HIT [57]. A single representative sequence was selected from each protein cluster and used for downstream analysis. Predicted proteomes of *M. larici-populina* and *P. graminis* f. sp. *tritici* were obtained from Duplessis et al. [13]. The secretomes were predicted as above for PST using PexFinder, followed by TMHMM and TargetP analysis. Proteins from the three predicted secretomes were then clustered based on sequence similarity using TribeMCL [26] following methods described in [58].

### Annotation of secreted protein tribes with effector features

Automated BlastP-based annotation was performed on proteins included in the secretome tribes using Blast2GO [59] with default parameters. In addition, BlastP analysis of proteins in the secretome tribes was conducted using the haustorial EST database constructed previously [12], with an e-value cutoff of 10^-5^. We searched each protein for the effector motifs [L/I]xAR [19,60], [R/K]CxxCx12H [19], RxLR [61], [Y/F/W]xC [62,63], YxSL[R/ K] [64] and G[I/F/Y][A/L/S/T]R [7] between amino acids 10 to 110 using Perl scripts. Nuclear localisation signals were predicted with PredictNLS [65]. Protein internal repeats were predicted using T-Reks [66]. We also assessed the length of the flanking intergenic regions (FIRs) between genes and the cysteine content of proteins using custom Perl scripts. Proteins cataloged as small and cysteine rich were less than 150 amino acids long and had a cysteine content higher than 3% as defined in Saunders et al. [12].

### Scoring secreted protein tribes for the likelihood of containing effector proteins

First, the method described in Saunders et al. [12] was used to assign an e-value to each feature within a tribe. The e-value was based on the number of proteins within a tribe that displayed a particular feature, relative to the likelihood of a tribe of the given size containing the same number of proteins with that particular feature by chance (Additional file 13). Next, the individual e-values were log converted into a score and a combined score calculated giving more weight to features that may be more indicative of effector proteins which included high expression in haustoria (a, in top 100 expressed genes; b, in top 500 expressed) or infected material (c, in top 100 expressed genes, d, in top 500 expressed), similarity to previously characterized HESPs or AVR effector proteins (e). A lower weight was given to other effector-related criteria that included the absence of any annotation (f) and the score associated with small cysteine-rich proteins (g).

$$\begin{array}{c} \mathrm{Combined}\;\mathrm{score}=\mathrm{round}\left(\log\left(a+b+c+d+e+1,2\right)\right)\\ \;*\left(1+0.1*\left(f+g\right)\right),3) \end{array}$$

The combined score was then used to rank the tribes based on their likelihood of containing potential effector proteins. The features associated with the top 100 ranked secreted protein tribes were visualized using Circos [67].

### qRT-PCR of effector candidates during infection

Wheat seedlings (cv Avocet ‘S’) were infected with PST-08/21 and incubated as described above. Six biological replicates were sampled at each time point (20 hours, 1 dpi, 6 dpi or 14 dpi). For each sample, 2.5 μg of total RNA was extracted and used for cDNA synthesis with the SuperScript First-Strand Synthesis System for RT-PCR (Invitrogen, UK). qRT-PCR was undertaken for 22 candidate effectors described in Additional file 15. Transcript levels were determined on a LightCycler® 480 instrument (Roche Applied Science, UK) using LightCycler 480 SYBR Green I Master (Roche) and the following conditions: 5 minutes at 95&z.ousco;C; 40 cycles of 15 sec at 95&z.ousco;C, 15 sec at 60&z.ousco;C, 20 sec at 72&z.ousco;C. The PCR amplification specificity was checked by dissociation curve analysis (from 60&z.ousco;C to 95&z.ousco;C). Transcript levels were normalized with *P. striiformis* elongation factor 1 [15,68] and linearised values determined using the 2^-ΔΔ*CT*^ method [69].

## Competing interest

The authors declare that they have no competing interests.

## Authors’ contributions

DC, DS and CU wrote the manuscript; VS, SK, JD contributed corrections and suggestions; DC, DM, DS performed the bioinformatic analysis; VS conducted the wet-lab experiments; RB and XC conducted pathology tests on PST isolates; DC, DS, CU analyzed the data; DC, VS, SK, JD, DS, CU conceived and designed the experiments. All authors read and approved the final manuscript.

## Supplementary Material

Additional file 1 Summary of raw and trimmed reads and assemblies of PST-21, 43, 130, 87/7, and 08/21 genomic DNA. Click here for file

Additional file 2 **Quality assessment of genome assembly using CEGMA for five PST isolates.** Of the 248 core eukaryotic genes (CEGs) 88.7% could be identified in the three PST US isolate genomes (PST-130, PST-43 and PST-21). The CEGMA pipeline distinguishes between CEGs found in complete (A) copies or as partial fragments (B) and separates the CEGs based on levels of conservation across higher eukaryotes, with group 4 being the most conserved. The levels of complete gene coverage were high for all US isolates, indicating few core eukaryotic genes were split across contigs. For the two UK isolates (PST-08/21 and PST-87/7) complete gene coverage was reduced compared to partial gene coverage, indicating higher levels of fragmentation for these genomes. Click here for file

Additional file 3 Variant calls in the pairwise comparisons between all PST isolates used in this study. Click here for file

Additional file 4 Summary of the reciprocal mapping of sequence reads for the 5 genomes using each in turn as a reference. Click here for file

Additional file 5 **Assessing the genetic diversity both within and between PST isolates.** A. Summary of the main steps used to identify genetic variants between PST isolates. B. Diagram to illustrate how we defined heterokaryotic (het) and homokaryotic (hom) variants between isolates. C. Illustration to show how synthetic genes were generated from homokaryotic SNPs identified in Illumina reads of isolate B mapped to the reference isolate A. Click here for file

Additional file 6 Number of SNPs identified in each gene of the 5 PST genomes. Click here for file

Additional file 7 Pairwise dN values calculated using Yn00 for each gene of the 5 PST genomes, comparing all isolates. Click here for file

Additional file 8 Pairwise dN/dS values calculated using Yn00 for each gene of the 5 PST genomes, comparing all isolates. Click here for file

Additional file 9 **(PST21_04206 displays sequence polymorphisms and positive selection between UK isolates and US PST-43 when compared to the synthetic gene from PST-130.** A. Sequence alignment of PST21_04206 and the synthetic genes that incorporate the SNP information from the other four isolates sequenced, illustrating sequence polymorphisms between isolates. Polymorphic residues are indicated below the sequence by red stars. B. Positive selection analysis on the PST21_04206 synthetic gene set demonstrated positive selection when US PST-43, UK PST-87/7 and PST-08/21 when compared against the US PST-130 sequence. Click here for file

Additional file 10 Depth, fold, and breath mapping coverage values for individual gene sequences that were identified as absent in any isolate. Click here for file

Additional file 11 Summary of RNAseq reads from PST-08/21 infected tissue and haustorial libraries 1 and 2. Click here for file

Additional file 12 **Raw and DESeq normalized Illumina counts of reads mapped onto each of the transcripts of the 5 PST genomes with the fold enrichments in the haustoria libraries and the associated *P*-values calculated using the DESeq statistical analysis.** Click here for file

Additional file 13 **Complete list of tribes with full annotation data and matching features.** The file contains (i) the list of proteins included in the tribe analysis with full annotation including effector properties, and (ii) the list of tribes with the number of proteins matching effector properties they contain. Click here for file

Additional file 14 Summary of the relative gene expression values measured using quantitative RT-PCR for 22 selected effector candidates. Click here for file

Additional file 15 Primers used in the quantitative RT-PCR experiments. Click here for file
