# Supplementary figures and images for: Genome analyses of the wheat yellow (stripe) rust pathogen Puccinia striiformis f. sp. tritici reveal polymorphic and haustorial expressed secreted proteins as candidate effectors
Source: BMC Genomics. 2013 Apr 22;14:270. doi: 10.1186/1471-2164-14-270 (PMC3640902; doi:10.1186/1471-2164-14-270)

**A**

Complete

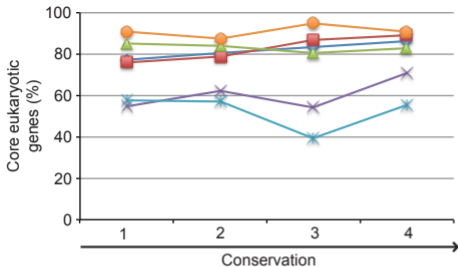**B**

Partial

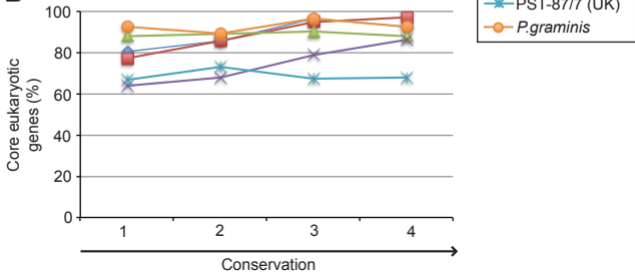

Supplement: Additional file 2 — Quality assessment of genome assembly using CEGMA for five PST isolates. Of the 248 core eukaryotic genes (CEGs) 88.7% could be identified in the three PST US isolate genomes (PST-130, PST-43 and PST-21). The CEGMA pipeline distinguishes between CEGs found in complete (A) copies or as partial fragments (B) and separates the CEGs based on levels of conservation across higher eukaryotes, with group 4 being the most conserved. The levels of complete gene coverage were high for all US isolates, indicating few core eukaryotic genes were split across contigs. For the two UK isolates (PST-08/21 and PST-87/7) complete gene coverage was reduced compared to partial gene coverage, indicating higher levels of fragmentation for these genomes. [file 1471-2164-14-270-S2.pdf]

# A

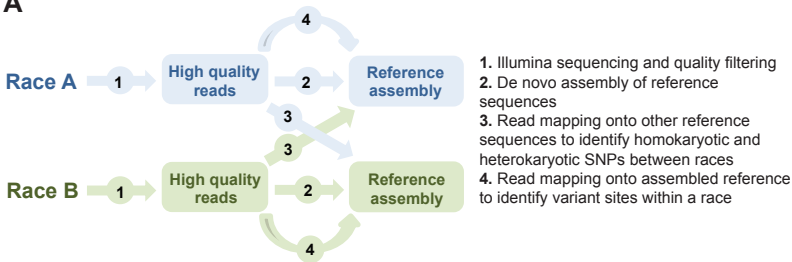

# B

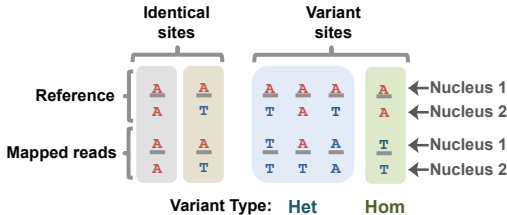

**C**

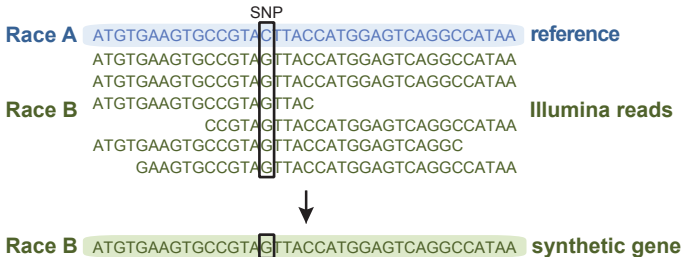

Supplement: Additional file 5 — Assessing the genetic diversity both within and between PST isolates. A. Summary of the main steps used to identify genetic variants between PST isolates. B. Diagram to illustrate how we defined heterokaryotic (het) and homokaryotic (hom) variants between isolates. C. Illustration to show how synthetic genes were generated from homokaryotic SNPs identified in Illumina reads of isolate B mapped to the reference isolate A. [file 1471-2164-14-270-S5.pdf]

**A**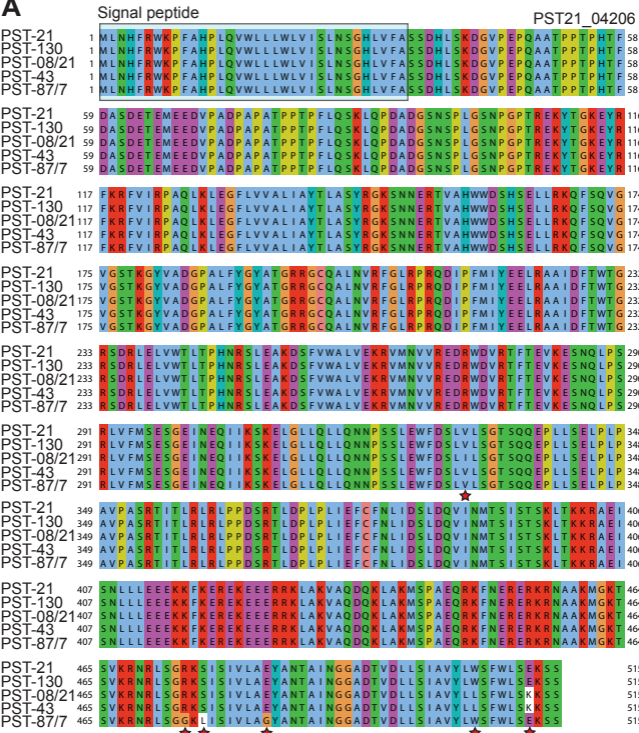**B**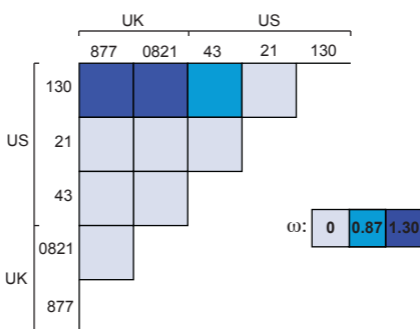

Supplement: Additional file 9 — (PST21_04206 displays sequence polymorphisms and positive selection between UK isolates and US PST-43 when compared to the synthetic gene from PST-130. A. Sequence alignment of PST21_04206 and the synthetic genes that incorporate the SNP information from the other four isolates sequenced, illustrating sequence polymorphisms between isolates. Polymorphic residues are indicated below the sequence by red stars. B. Positive selection analysis on the PST21_04206 synthetic gene set demonstrated positive selection when US PST-43, UK PST-87/7 and PST-08/21 when compared against the US PST-130 sequence. [file 1471-2164-14-270-S9.pdf]
