# Supplementary material for: Genome analyses of the wheat yellow (stripe) rust pathogen Puccinia striiformis f. sp. tritici reveal polymorphic and haustorial expressed secreted proteins as candidate effectors
Source: BMC Genomics. 2013 Apr 22;14:270. doi: 10.1186/1471-2164-14-270 (PMC3640902; doi:10.1186/1471-2164-14-270)
Supplement: Additional file 15 — Primers used in the quantitative RT-PCR experiments. [file 1471-2164-14-270-S15.docx]

Additional file 15. Primers used in the qRT-PCR experiments.

| **Gene ID** | **Tribe no.** | **Forward primer sequence (5'->3')** | **Reverse primer sequence (5'->3')** | **Amplicon size (bp)** |
| --- | --- | --- | --- | --- |
| PST0821_02418 | 413 | TTGTCCCGAAAGTGACTGTG | TTTGCTTTGTCAAAATCTCACAC | 122 |
| PST21_13335 | 413 | GATTGGACTCCCACAAAAGC | TCGACATTCTCCTTTCATCTG | 63 |
| PST43_18397 | 276 | CGAGCTGCAATGAGTATCCA | GGTTTTCACCGGTTGTGACT | 76 |
| PST21_11390 | 6 | CCAGGTTGAAATGGTGACCT | TGCACAAAGATGCATACAAAAA | 109 |
| PST877_03650 | 6 | CGTTTACACACAGGGAAAAGG | GGGAAACAAGGTCACATCAAA | 215 |
| PST0821_05825 | 6 | GGGCGTGTTAGACATCCAGA | GAGATCAAAGCCCACAAAGG | 164 |
| PST21_18221 | 238 | TAGCAGGCGGTGGTGATT | GCCTGTACATCTACTTGGGATTG | 154 |
| PST21_18220 | 238 | GCCCTGATGAAGAGAGAGCTT | TTGCTCCACCTCTGAATGCT | 73 |
| PST130_12532 | 308 | ACGTCAAGTTGTCGAGGTTG | TGCAGTCTTGGTGGCTTCTA | 123 |
| PST43_17026 | 351 | GGTACTGAAGAACCCAAAGACG | CCCAGGCTTGTCGAAGTTAC | 79 |
| PST130_10194 | 79 | TATCGTCCAAGGGGGAGGTA | AAGGGCTTGAACAATTCTGG | 300 |
| PST0821_05302 | 593 | TGGTTATCGGCTTACTAATTGC | ATGCCTGAAAGGTCGTTGTC | 133 |
| PST43_13507 | 467 | GCTGATCCGAAGACTGGTTT | AGTATCGGGAAAAGGGGAAA | 83 |
| PST43_01959 | 426 | CGGTTACTGAAGTTGTGAAAGA | ATCTAATGCCTCGGCCTTCT | 98 |
| PST21_09249 | 1232 | TGTCGCGAGCTTACTGATTG | TGTAAGCAGGTCGAGCCATT | 65 |
| PST0821_06496 | 1004 | CTATGGCTCAAGCACCCTCA | AGCGTCCGCGATTACAAAC | 128 |
| PST21_13514 | 928 | CGCAGTGATGATTGAGGT | AAACTTGTTCGGTGGATG | 188 |
| PST43_18806 | 544 | GCACCGATGATACCAAAG | AGAAATACGAGGCGAAGAT | 246 |
| PST877_15584 | 1118 | TACTTCCCCGTGCCATTAT | ACATCTACACCACATAACTCAACAG | 140 |
| PST21_16369 | 596 | ATGGAAGGGACCGAGGAGG | GTGGCAGCAATGGCAACG | 227 |
| PST21_18431 | 317 | GAGAGCGAGAAAGCATGGAGAC | ACCGAGACTTGGCAGCTAACC | 159 |
| PST43_08077 | 544 | CCGCTGGAAGTGTTGATGG | TCTTGATAGGGTCCGTGCTGT | 180 |
| PST-EF1^a^ | -- | TTCGCCGTCCGTGATATGAGACAA | ATGCGTATCATGGTGGTGGAGTGA | 159 |

^a^ PST *elongation factor 1* (PST-EF1) was used as the reference gene (Ling et al 2007)
